# Selective Internal Radiation Therapy with Yttrium-90 for Intrahepatic Cholangiocarcinoma: A Systematic Review on Post-Treatment Dosimetry and Concomitant Chemotherapy

**DOI:** 10.3390/curroncol29060306

**Published:** 2022-05-24

**Authors:** Sedighe Hosseini Shabanan, Nariman Nezami, Mohamed E. Abdelsalam, Rahul Anil Sheth, Bruno C. Odisio, Armeen Mahvash, Peiman Habibollahi

**Affiliations:** 1 Department of Radiology, University of California San Diego, San Diego, CA 92093, USA; shosseinishabanan@ucsd.edu; 2 Division of Vascular and Interventional Radiology, Department of Diagnostic Radiology and Nuclear Medicine, University of Maryland School of Medicine, Baltimore, MD 21201, USA; nariman.nezami@umm.edu; 3 Experimental Therapeutics Program, University of Maryland Marlene and Stewart Greenebaum Comprehensive Cancer Center, Baltimore, MD 21201, USA; 4 Department of Interventional Radiology, Division of Diagnostic Imaging, The University of Texas MD Anderson Cancer Center, Houston, TX 77030, USA; meabdelsalam@mdanderson.org (M.E.A.); rasheth@mdanderson.org (R.A.S.); bcodisio@mdanderson.org (B.C.O.); armeen.mahvash@mdanderson.org (A.M.)

**Keywords:** selective internal radiation therapy, cholangiocarcinoma, dosimetry, microsphere, concomitant chemotherapy

## Abstract

Selective internal radiation therapy (SIRT) with yttrium-90 (^90^Y)-loaded microspheres is increasingly used for the treatment of Intrahepatic Cholangiocarcinoma (ICC). Dosimetry verifications post-treatment are required for a valid assessment of any dose-response relationship. We performed a systematic review of the literature to determine how often clinics conducted post-treatment dosimetry verification to measure the actual radiation doses delivered to the tumor and to the normal liver in patients who underwent SIRT for ICC, and also to explore the corresponding dose-response relationship. We also investigated other factors that potentially affect treatment outcomes, including the type of microspheres used and concomitant chemotherapy. Out of the final 47 studies that entered our study, only four papers included post-treatment dosimetry studies after SIRT to quantitatively assess the radiation doses delivered. No study showed that one microsphere type provided a benefit over another, one study demonstrated better imaging-based response rates associated with the use of glass-based TheraSpheres, and two studies found similar toxicity profiles for different types of microspheres. Gemcitabine and cisplatin were the most common chemotherapeutic drugs for concomitant administration with SIRT. Future studies of SIRT for ICC should include dosimetry to optimize treatment planning and post-treatment radiation dosage measurements in order to reliably predict patient responses and liver toxicity.

## 1. Introduction

Cholangiocarcinoma is the second most common primary hepatic malignancy, and over the past several decades its incidence has increased in the United States and worldwide [1]. Surgical resection and liver transplantation are the only cures for intrahepatic cholangiocarcinoma (ICC); however, most patients are diagnosed with advanced-stage ICC, for which curative surgery is impossible [2]. In addition, more than 50% of patients with early-stage ICC who undergo surgical resection experience disease recurrence after a median of 20 months [3]. In clinical practice, various locoregional therapies are used to treat hepatic tumors; these include thermal liver ablation methods (radiofrequency or microwave), external beam radiation therapy, and trans-arterial therapies such as hepatic artery infusion, chemoembolization, and radioembolization [4,5]. Trans-arterial radio-embolization, or selective internal radiation therapy (SIRT) with yttrium-90 (^90^Y), was first introduced in 1965 [6] and has evolved to become a treatment option for unresectable primary or metastatic hepatic tumors. SIRT may be used as a first-line treatment for select patients, as an adjunct to systemic chemotherapy, or after the failure of other therapies [7]. Newer generations of systemic therapies, including immunotherapeutic agents, may also be considered to be concomitantly administered with SIRT in future research [8,9].

The liver is a radiosensitive organ, and the radiation dose required to destroy hepatic tumors is greater than the threshold dose of the normal hepatic parenchyma [10]. In view of this, the liver’s dual blood supply, as well as the differential blood flow to the tumor versus the normal liver parenchyma, provided the rationale for SIRT. In SIRT, ^90^Y microspheres are injected into the hepatic artery, the main supply for liver tumors; the normal liver parenchyma that receives blood from both the portal and systemic circulation is partially spared [11]. In addition to the differential blood supply, the average penetration depth of 2.5 mm for the high-energy beta radiation from ^90^Y-loaded microspheres further helps to achieve radiation demarcation between the tumor and the liver parenchyma [12].

Most studies on the application of SIRT in ICC presume uniform distribution of ^90^Y microparticles and perform treatment planning to deliver a mean absorbed dose of 120 ± 20 Gy to the tumor and a threshold dose of no more than 50–70 Gy to the normal liver parenchyma [13]. Historically radioembolization planning employed several dosimetry models, including Body Surface Area Method (activity calculation), the Single Compartment Medical Internal Radiation Dose, and the Partition Model [14], to estimate the absorbed dose to the tumor and normal liver. Models of activity prescription have substantially advanced, with the partition dosimetry model providing the most personalized patient-specific model for predicting radiation uptake in normal and tumoral tissue by using ^99^mTc-MAA SPECT-CT as a surrogate for ^90^Y imaging [11] and considering the extrahepatic deposition of radioactivity [15]. Relying only upon pretreatment radiation dose calculations could cause clinicians to overlook the biological parameters affecting treatment outcomes [16]. Moreover, several studies have demonstrated considerable discrepancies between pretreatment predictive planning and dosimetry verification studies after radioembolization [15,17,18]. These discrepancies possibly originate from variations in the flow dynamics, catheter position and the sizes, weights, and densities when comparing ^99^mTc-MAA particles and ^90^Y microspheres [18]. Performing verification imaging studies after SIRT is essential for a precise assessment of the activity distribution and the dose delivery, for dose-response and toxicity studies, and for the clinical management of extrahepatic deposition [13]. Moreover, despite nearly three decades of clinical SIRT use, there is no consensus on the optimal dosing for disease control, and few studies have examined the dose-response relationship for SIRT in patients with ICC and it is not clear whether they measured the true, delivered radiation dose through post-treatment dosimetry studies. Therefore, in the present study, we systematically reviewed the literature on the application of SIRT in ICC to investigate how often clinics conducted treatment verification studies after treatment in order to measure the actual radiation doses delivered to the tumor and to the normal liver. It was also investigated whether, in those studies with true post-treatment dosimetry verification, a tumor dose threshold is predictive of tumor response, or a normal liver dose threshold is predictive of hepatic toxicity. We also looked into other factors that can potentially influence treatment outcomes, including the microsphere types and the application of concomitant chemotherapy.

## 2. Material and Methods

The study was conducted according to the Preferred Reporting Items for Systemic Reviews and Meta-Analyses (PRISMA) guidelines for systematic reviews [19]. A systematic literature review of English-language journal articles and conference abstracts on the application of SIRT in ICC patients was conducted using the PubMed, Embase, and Scopus databases. Articles and abstracts published on or before 10 February 2022, were included. A search query was developed following a review of the search strategies used in published systematic reviews in the same field [20,21]. The following Medical Subject Heading Terms were combined: “yttrium radioisotopes”, “radiopharmaceuticals”, “embolization”, “therapeutic”, “cholangiocarcinoma”, and “bile duct neoplasms”. We also searched for “radioembolization”, “locoregional therapy”, and “liver tumor”.

One reviewer (SHS) screened titles, abstracts, and keywords to exclude irrelevant papers. Two independent reviewers (SHS, PH) then performed full-text assessments against the inclusion and exclusion criteria to identify all journal articles and conference abstracts reporting the use of SIRT in at least one patient with ICC. In the event of a disagreement between two reviewers, a mutual dialogue was convened to resolve the issue. Studies that included ICC patients but reported only unified results for all the examined tumor types were excluded, as were studies that did not have radiation dose information or whose complete results were provided in another published paper. Studies of SIRT in patients with combined hepatocellular carcinoma and ICC histo-pathologies with unified results, as well as case reports, comments, and editorials, were also excluded. Studies with different patient populations were considered to be included in this study when SIRT was used as a first-line treatment or following the failure of other treatments. In addition, in some studies, a mixed population of both treatment-naïve and refractory patients received SIRT, and unified results were reported, which were also included in this review.

Data extracted from the eligible studies were entered into data extraction tables, including the following:

Study characteristics (i.e., authors, year of publication, study design including retrospective versus prospective), sample size, the patient population in terms of previous treatments tried (treatment-naïve and/or refractory); the ^90^Y-treatment specifications (e.g., type of ^90^Y microspheres administered, number of treatment courses in case of repeated procedures, concurrent chemotherapy, imaging modality used for post-treatment dosimetry), and treatment outcomes (e.g., imaging-based response assessment, the timing of imaging-based response, overall survival (OS), and progression-free survival (PFS)).

## 3. Results

**Figure 1:**
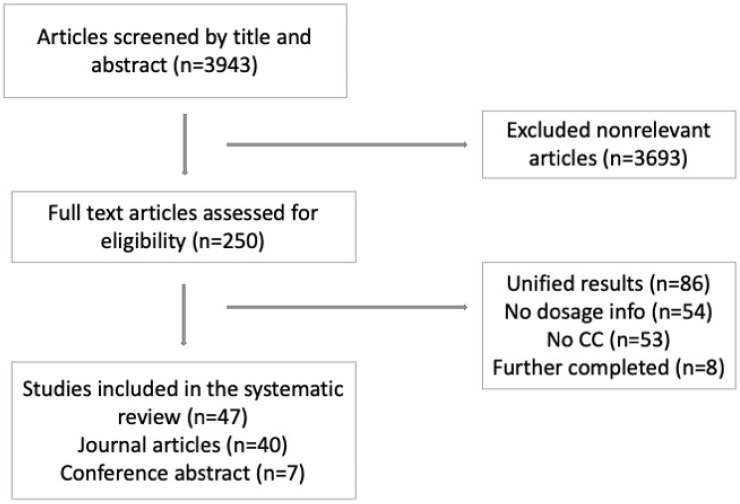
Identification of included papers. ICC: Intrahepatic Cholangiocarcinoma.

A total of 3943 papers were identified for screening. We excluded 3693 irrelevant papers based on their titles, abstracts, and/or keywords, leaving 250 papers that underwent full-text assessment (Figure 1). After implementing our exclusion criteria, 47 papers were eligible for data extraction. Of the final 47 included papers, 40 were journal articles [22,23,24,25,26,27,28,29,30,31,32,33,34,35,36,37,38,39,40,41,42,43,44,45,46,47,48,49,50,51,52,53,54,55,56,57,58,59,60,61] (Table 1), and seven were conference abstracts [62,63,64,65,66,67,68] (Table 2).

Thirty-two journal articles and five conference abstracts had a retrospective design, and eight journal articles and two conference abstracts were prospective studies. Twenty-eight studies used SIR-Spheres, nine used TheraSpheres, nine used both, and one conference abstract did not specify the type of ^90^Y microspheres used [64]. In addition, five papers compared SIRT outcomes for patients treated with the two types of microspheres [27,28,34,41,43].

Thirty studies included both treatment-naïve patients and those with the recurrent disease following other treatments, five studies assessed only outcomes following SIRT as a first-line therapy [29,42,43,44,54] and nine studies included only patients in whom prior treatments had failed [22,24,27,34,38,47,55,56,66]. The remaining three studies did not provide details on their patient populations [62,64,68].

The clinical outcome of SIRT was mainly reported as the OS, which was not statistically reached in three studies [29,54,64]. Some studies recorded PFS, liver-specific PFS, and the time to disease progression (Table 1 and Table 2). For tumor response assessment, different sets of imaging response criteria were used in published studies, including the Response Evaluation Criteria in Solid Tumors [RECIST], modified RECIST [mRECIST], Positron Emission Tomography Response Criteria in Solid Tumors [PERCIST], European Association for the Study of Liver Disease [EASL], and World Health Organization (WHO) classification.

In 11 studies, SIRT and chemotherapy were given concomitantly to at least one patient (Table 3). One study was excluded from our review because it provided unified dosimetry results for two different malignancies in their patient population, ICC and pancreatic cancer; however, related information regarding the concomitant chemotherapy was added to Table 3 [69].

Eleven studies described the use of imaging to assess the distribution of ^90^Y microparticles in the liver after SIRT. Of these, seven used Bremsstrahlung ^90^Y SPECT-CT [23,24,26,28,41,46,49], and four used ^90^Y positron emission tomography (PET)-CT [31,32,42,45] to confirm the distribution of ^90^Y microspheres in the lesions and exclude extrahepatic deposition. True dosimetry verification studies were performed in only four studies (Table 4) [22,25,27,43].

Regarding the dose-response association based on true post-treatment dosimetry, Willowson et al. [22] conducted a lesion-based study on 18 patients with ICC treated with resin microspheres, using ^90^Y PET-CT and ^18^F-FDG PET-CT for dose and response assessments, respectively. They defined lesion response as total lesion glycolysis reduction of at least 50% and found that there was a trend for a dose-response relationship with a higher tumor average dose in responding lesions, although it did not meet statistical significance (*p* = 0.29). Cheng et al. [27] considered objective response rate, defined as the pooled, 3-month, mRECIST-based, complete and partial response rates, as the tumor response and measured the tumor delivered radiation dose using ^90^Y SPECT-CT. Their study demonstrated that a threshold tumor dose of 78.9 and 254.7 Gy for resin and glass microspheres, respectively, can predict tumor response with 80% specificity. In terms of survival analysis, tumor dose cutoff points of ≥75 Gy for resin and ≥150 Gy for glass, were substantially associated with a longer OS. Tumor dose was also demonstrated to positively affect TTP in a study by Depalo et al. [25], using ^90^Y PET-CT for dosimetry. Based on their calculations, an average dose of 180 Gy in resin-based SIRT, was required to achieve partial tumor response, as defined by RECIST 1.1, at three months after the treatment. The authors of the other article, reporting a retrospective study on a sample size of five patients treated with glass, and five patients with resin microspheres, performed ^90^Y SPECT-CT–based dosimetry after SIRT and calculated mean tumor doses of 205.7 ± 19.7 Gy and 128.9 ± 10.6 Gy for glass- and resin-based microspheres, respectively (*p* < 0.001) [43]. However, their study had a very small sample size and lacked a survival-based dose-response analysis and follow-up imaging studies to show responses to treatment.

In terms of toxicity, three studies investigated potential variations based on the administered microsphere and found that glass-based and resin-based microspheres have similar toxicity profiles [27,34,43], of which true post-treatment dosimetry was performed in two studies [27,43]. In one study, the mean absorbed ^90^Y dose in the normal liver tissue was significantly lower with the use of glass microspheres than the resin (42.4 ± 4.5 Gy and 53.6 ± 4.3 Gy, respectively; *p* < 0.001), and the tumor-to-normal liver dose ratio was subsequently higher with glass-based spheres (*p* < 0.001) [43]. Their study results may suggest that glass-based radioembolization can deliver a higher radiation dose to a tumor without a remarkable dose increase to the normal liver parenchyma, lowering the risk of treatment-induced toxicity. However, clinical and laboratory toxicity rates were similar in both groups. In the other study with post-treatment dosimetry, the tumor-to-normal liver ratio was not statistically different between glass and resin-based SIRTs (*p* = 0.24) as the clinical and laboratory toxicity rates were the same between groups [27]. None of the studies with true post-treatment dosimetry determined a normal liver dose threshold to predict treatment-induced toxicity.

## 4. Discussion

### 4.1. Dosimetry and Dose-Response Relationship

Our systematic review showed that most published studies of SIRT in ICC patients reported only a nonspecific mean administered radiation dose based on pretreatment prediction models and did not provide any information on the actual tumor dose based on post-treatment imaging. Most studies that carry out post-treatment verification dosimetry after SIRT include patients with hepatocellular carcinoma because ICC is comparatively rare. As presented in Table 4. we could only identify four published papers conducting a dosimetry study after SIRT. There were two imaging modalities used in verification studies, Bremsstrahlung ^90^Y SPECT-CT imaging and ^90^Y PET-CT. The main factor that limits the clinical application of treatment verification using Bremsstrahlung ^90^Y SPECT-CT stems from the characteristics of ^90^Y, which lacks a distinct photopeak and thus distorts the image resolution [70]. Several measures have been taken to enhance the quality of these images, such as using certain collimators or applying scatter and attenuation correction methods, but the use of these methods is restricted in practice because of the technical issues involved and the meticulous calibration required [11]. At our institution, ^90^Y SPECT-CT after SIRT has been standard for years. However, some experts believe that ^90^Y PET-CT has a better spatial resolution with lower scatter [71], and the most recent international guidelines for SIRT for patients with liver malignancies recommend the use of ^90^Y PET-CT in dosimetry studies after SIRT [13]. A study is underway at our institution comparing the accuracy of ^90^Y SPECT-CT versus ^90^Y PET-CT after SIRT, which will provide more information on this matter.

Out of the three studies that investigated the dose-response relationship based on true post-treatment dosimetry (Table 4), Willowson et al. [22] could not certify any statistically significant dose-response association, although their study showed that metabolically responsive lesions had higher average tumor doses. Depalo et al. [25] proved that a larger mean delivered tumor dose is significantly associated with longer TTP, although no certain dose threshold was calculated. Cheng et al. [27] considered a mean tumor dose of 75 Gy for resin microspheres as a cutoff point predictive of remarkably longer OS. In terms of response assessments based on imaging-based criteria, Depalo et al. [25] found a mean tumor dose threshold of 180 Gy is associated with a better partial response, and Cheng et al. [27] measured a threshold mean tumor dose of 78.9 Gy for a significantly higher rates of complete and/or partial response, both with resin-based treatments. Besides heterogeneities in their patient populations, the application of two different modalities for dosimetry could possibly justify the variations in tumor dose cutoffs for an imaging-based response. Different dose thresholds were measured for glass-based procedures, which are further explained in the following section.

### 4.2. Types of ^90^Y Microspheres

Currently, two types of ^90^Y microspheres have been approved for clinical use in the US: SIR-Spheres (SIRTex Medical, Sydney, Australia), which are ^90^Y-coated resin microspheres, and TheraSpheres (Therasphere BTG, Ontario, Canada), which are insoluble, glass microspheres embedded with ^90^Y. The radiation doses of these two microspheres vary because of their different specific activities, specific densities, and particle sizes. The specific activity of glass-based TheraSpheres (~2500 Bq per sphere) is much higher than that of resin-based SIR-Spheres (50 Bq per sphere); therefore, many more resin microspheres are needed to deposit the same level of activity achieved with just a few glass microspheres. This may explain SIR-Spheres’ higher rates of vascular stasis [11]. However, on the basis of our experience, we firmly believe that the tumor response is not only related to the actual delivered dose of radiation but also the number of delivered microspheres per tumor volume and the homogeneity of microsphere distribution in tumor tissue. This viewpoint is justified by the very short penetration depth of high energy beta radiation from the ^90^Y isotope, as it has been demonstrated that around 90% of the radiation dose is delivered in a radial distance of 300 μm around ^90^Y microspheres [72].

This review included 28 studies that used resin-based microspheres and nine that used glass-based microspheres for the treatment of patients with ICC. Nine studies used both types of microspheres, and one study did not report the type of microspheres used (Table 1 and Table 2). As expected, in those studies that used both types of microspheres, the mean or median activity or radiation dose administered using glass-based microspheres was higher than that administered using resin. In those publications with post-treatment dosimetry studies, the mean delivered tumor dose was around 200 to 250 Gy for glass-based and 80 to 130 Gy for resin-based treatments (Table 4).

In terms of efficacy, Shaker et al. [41] found a clinically, though not statistically, significant difference in PFS between patients treated with resin-based microspheres and those treated with glass-based microspheres (15.6 months vs. 2.4 months; *p* = 0.46). Three other studies compared the survival outcome of the SIRT by the type of microsphere used, in none of which a statistically significant difference could be reached in median OS, PFS, or liver-specific PFS [27,28,34]. Imaging-based response assessment carried out by Buettner et al. [34], demonstrated that glass-based microspheres elicited a higher rate of partial response (as determined using RECIST at 6 months after the SIRT) than resin-based microspheres (*p* = 0.008); Still, their study lacked a true post-treatment dosimetry verification study. There were only two studies that performed a post-SIRT dosimetry study comparing the TheraSpheres and SIR-Spheres, among which only one recent paper by Cheng et al. [27] carried out clinical and radiological outcome analyses, and the other one did not investigate any long-term outcome of the treatment [43]. Cheng et al. [27] found that patients treated with glass-based microspheres have a slightly higher objective response rates, still not statistically significant (*p* = 0.47), as compared to those receiving resin-based microspheres, using the mRECIST three months after the procedure. In terms of toxicity, three studies investigated potential variations based on the microsphere type and found that glass-based and resin-based microspheres have similar toxicity profiles and no specific normal liver dose threshold was determined to cause clinical or laboratory toxicities [27,34,43]. These results are compatible with the results of a pooled analysis by Zhen et al. [73], which found a comparable outcome of the SIRT employing both microsphere types, with a median overall survival of around 14 months and disease control rate of 77% for both groups. However, it is noteworthy that most of the studies comparing these two groups lack post-treatment dosimetry data and further investigations, including retrospective and prospective studies with larger patient populations and post-treatment verification studies, are required to determine whether either type of microsphere provides more benefit in patients with ICC.

### 4.3. Concomitant Chemotherapy

The mainstay of treatment for cancers originating in the biliary tract, including ICC, is systemic chemotherapy with gemcitabine-based regimens [74]. Cisplatin has also been established to provide survival benefits when combined with gemcitabine [75]. Although it has been defined variously, concomitant chemotherapy is routinely referred to as the administration of chemotherapy no more than three months prior to SIRT [54], which can potentially provide a more prolonged survival than SIRT alone because of the radio-sensitizing effects of gemcitabine [76]. In a meta-regression study by Cucchetti et al. [7], the pooled median survival duration was 19.5 months for patients who received SIRT and concomitant chemotherapy but only 5.5 months for patients who received SIRT alone, which supports the concomitant use of systemic chemotherapy with SIRT in patients with ICC. The authors also found that the 2-year survival rate of the patients who received the combination treatment (42.5%) was significantly higher than that of the patients who received SIRT alone (<10%; *p* = 0.04). However, only one of every five studies included in their analysis had patients whose treatment met the aforementioned definition of concomitant chemotherapy [54].

Among the papers included in the present study, 11 reported studies in which at least one patient received concomitant chemotherapy (Table 3). In a study by Manceau et al. [44], treatment-naïve patients received one of the three different chemotherapy regimens: gemcitabine plus cisplatin, cisplatin plus fluorouracil, or gemcitabine plus oxaliplatin. They reported a 3-month response rate of 69% and successful downstaging in 49% of the patients. Edeline et al. [33] assessed the outcome of concomitant chemotherapy with gemcitabine and cisplatin in treatment-naïve patients in a phase II clinical trial. They reported a 3-month objective response rate of 39% and a disease control rate of 98% in 40 of 41 patients, as well as downstaging in 22% of patients. For safety, the gemcitabine dose is usually reduced to 300 mg/m^2^—the recommended dose for patients with pancreatic cancer undergoing gemcitabine-based chemotherapy concomitantly used with SIRT [77]. However, in a recent phase Ib clinical trial, in which escalating doses of gemcitabine were given concomitantly with SIRT to five treatment-naïve patients with ICC, doses of up to 600 mg/m^2^ could be administered safely, with only transient liver toxicity [69]. This study was excluded from our review because it provided unified dosimetry results for two different malignancies of ICC and pancreatic cancer in their patient population, however the study’s chemotherapy-related information is included in Table 3. Despite what has been widely accepted on the effectiveness of concomitant chemotherapy, a recent study by Depalo et al. [25] could not prove the radio-sensitizing effects of concomitant chemotherapy; however, their study was limited by the very few numbers of heterogenous patients with a wide standard deviation of means. More studies are warranted to establish optimal chemotherapy doses and to confirm response rates following SIRT with concomitant chemotherapy.

### 4.4. Treatment Outcome

Many studies investigated the ICC response to the SIRT using different imaging response criteria (Table 1 and Table 2). In a recent, pooled meta-analysis of 14 papers and 608 patients by Mosconi et al. [78], the imaging-based objective response rate (as defined using RECIST at six months), was 19.3%. At least two other meta-analyses have reported imaging-based response assessments. The first, by Boehm et al. [20], reported an objective response rate of 27.4%, found in an analysis of five papers [56,58,59,60,61]. A more complete study by Al-Adra et al. [21], which had three papers in common with the study by Boehm et al., reported a 3-month partial response rate of 28% and a stable disease rate of 54%. Regarding the clinical outcomes of radioembolization in patients with ICC, Mosconi et al. [78] reported a median survival duration of about 13.5 months after treatment. Similar results were obtained in another meta-analysis by Boehm et al. [20] in 2015, which reported a median survival duration of 13.9 months. The publications we included in our study reported a wide range of median OS durations, ranging from 5.7 to 33.6 months [41,52]. The studies’ varied response rates possibly stem from differences in their patient populations and a lack of standardized tumor dosimetry. Moreover, several issues should be taken into consideration when interpreting the results of the survival analysis following radioembolization, including the type of ICC, staging [26], and pathological grading [45]. In 2010, Saxena et al. [60] reported that patients with mass-forming peripheral ICCs have a longer median survival duration than do patients with infiltrative lesions (18.3 months vs. 4.5 months). Similar results were also reported in a recent study by Paz-Fumagalli et al. [26], indicating that patients with mass-forming tumors had a longer survival (*p* = 0.002). Furthermore, as shown in several studies included in the present review, patient survival varies according to whether SIRT was used as a first-line treatment (for treatment-naïve ICC patients), for chemotherapy-refractory disease, or after the failure of other therapies [23,28,31,35,51]. In a meta-analysis by Cucchetti et al. [7], the median survival duration was about 24 months for treatment-naïve patients but only 11.5 months for patients with the chemotherapy-refractory disease (*p* = 0.048).

## 5. Conclusions

This study elucidates the gap in our current knowledge of SIRT in ICC. Post-treatment dosimetry is essential to verify the agreement between the intended and delivered radiation doses and also to investigate dose-response associations which could be carried out using ^90^Y PET-CT or ^90^Y Bremsstrahlung SPECT-CT as an alternative. According to our study results, there are very few publications available that have investigated dose-response relationship based on true post-treatment dosimetry, and in these studies, no consistent dose thresholds were established.

It should be noted that studies included in our systematic review had very heterogeneous patient populations in terms of concurrent or prior therapies other than SIRT. Refractory cases of ICC have a different prognosis from treatment-naïve patients, and interpreting the results as a unified matter may be biased. Moreover, studies comparing the efficacy and toxicity of SIRT based on the microsphere’s type were also limited by the shortage of patient populations.

The results of our work warrant further studies to conduct post-treatment dosimetry verification after the SIRT in order to reach a consensus regarding the tumor dose threshold needed to obtain an optimal response and the normal liver dose threshold related to treatment-induced toxicity.

## Figures and Tables

**Table 1:** Journal articles included in the study.

Author (Year), Type of Study	Patient Population	Radiation Dosage, Gy	Activity, GBq	Microsphere Type: Number of Patients Treated	RECIST, WHO, EASL at 1st Assessment	Survival Outcomes	Follow-Up Information	Notes and Other Findings
Willowson KP, et al. (2021) RS [22]	18 pts 23 SIRTs (5 pts with multiple SIRTs) Refractory	-	Median: 1.5 Mean: 1.62	Resin: 18	-	-	-	Lesion-based analysis with ^18^F-FDG PET/CT was done.
Sarwar, A. et al. (2021) RS [23]	31 pts 40 SIRTs (7 pts with multiple SIRTs) Mixed ^†^	Median: 150	Median: 1.9	Resin: 31	RECIST 1.1 at 2–3 mo (29 pts) CR:0, PR: 17.24%, SD: 68.96%, PD: 13.79% RECIST 1.1 at 6 mo (21 pts) CR: 0, PR: 23.8%, SD: 61.9%, PD: 14.28%	Median OS: 22 mo Median PFS: 5.4 mo Median TTP: 6.3 mo	Median FU: 14 mo 15 deaths	Post-SIRT ^90^Y SPECT-CT done qualitatively. Higher PFS in treatment-naïve vs. refractory patients, 7.4 vs. 2.7 mo (*p* = 0.00) as well as the TTP: 13 vs. 3 mo (*p* = 0.00)
Paprottka, K. J. et al. (2021) RS [24]	73 pts 103 SIRTs (6 pts with multiple SIRTs) Refractory	-	Median: 1.5	Resin: 73	RECIST at 3 mo CR: 0 PR: 24.65% SD: 49.31% PD: 26.02%	Median OS: 11.8 mo Mean OS: 18.9 mo Median PFS: 6.4 mo Mean PFS: 10.1 mo	-	Post-SIRT ^90^Y SPECT-CT done qualitatively. Median PFS higher with multiple SIRTs 24.4 vs. 5.8 mo (*p* = 0.04)
Depalo, T. et al. (2021) RS [25]	15 pts 21 SIRTs (Number of pts with multiple SIRT treatments unspecified) Mixed	Mean TD: 93 LD: 42	Mean: 1.16	Resin: 15	RECIST 1.1 at 3 mo CR: 0 PR: 20% SD: 40% PD: 40%	Median TTP: 7.3 mo	-	Tumor absorbed dose showed positive effect on TTP (*p* = 0.05). No difference found in radio-sensitivity (α parameter) in SIRT + concomitant chemo vs. SIRT alone.
Paz-Fumagalli, R. et al. (2021) RS [26]	28 pts 37 SIRTs (5 pts with multiple SIRTs) Mixed	Median: 256.8	Mean: 2.53	Glass: 28	mRECIST at 3 mo (25 pts) CR: 44.1% PR: 50% SD: 2.9% PD: 2.9% Response rate: 94.1% Control Rate: 97.1%	Median OS not reached. Median PFS: 8.8. mo OS in 3yrs: 59% PFS in 3yrs: 25%	Median FU: 13.4 mo 9 deaths	Post-SIRT ^90^Y SPECT-CT done qualitatively. Multifocal, bilobar, and larger tumors had a worse PFS (*p* = 0.00, *p* = 0.00, *p* = 0.04). Mass-forming tumors had a longer OS (*p* = 0.002)
Cheng B, et al. (2021) RS [27]	38 pts 45 SIRTs (Number of pts with multiple SIRT treatments unspecified) Refractory	Mean TD Resin: 78.9 Glass: 254.7	-	Resin: 18 Glass: 20	mRECIST at 3 mo CR: 15.78%, PR: 39.47%, SD: 23.68%, PD: 21.10% OR: Glass: 50% Resin: 61.11% (*p* = 0.47)	Median OS: Resin: 11.2 mo Glass: 10.9 mo (*p* = 0.54)	-	Dose response study is done. Microsphere type had no effect on survival for Resin 11.2 mo vs. Glass 10.9 mo (*p* = 0.54). Glass and resin had a similar toxicity profile.
Bozkurt, M. et al. (2021) RS [28]	19 pts * 24 SIRT (5 with multiple SIRT treatments) mixed	-	Mean Glass: 3.4 resin: 1.0 (*p* = 0.03)	Resin: 11 Glass: 13	RECIST 1.1 at unspecified time: CR: 7.7% PR: 15.4% SD: 30.8% PD: 46.2%	Mean OS: 11.1 mo resin: 8.6 mo glass: 10.1 mo (*p* = 0.63)	-	^90^Y SPECT-CT done qualitatively after SIRT. OS not different for naïve vs. refractory cases (*p* = 0.47)
Riby, D. et al. (2020) RS [29]	19 pts naïve	Median TD: 258 Median NTD: 73.4	Median: 1.9	Resin: 19	RECIST 1.1 at 3–6 mo (No separate results for SIRT cases)	Median OS not reached. Median RFS: 18.5 mo	Median FU: 44.0 mo	SIRT was applied to downstage the disease for surgical resection.
Mosconi, C. et al. (2020) RS [30]	55 pts mixed	-	Median: 1.2	Resin: 55	RECIST 1.1 at unspecified time (53 pts): CR: 7.6% PR: 49.1% SD: 35.9% PD:7.6%	Median OS: 16.7 mo Median PFS: 6 mo	Median FU: 12.5 mo	Two pts died within 3 mo
Köhler, M. et al. (2020) RS [31]	46 pts mixed	-	Median: 1.7	Resin: 46	RECIST 1.1 at 3 mo (44 pts): CR: 0 PR: 34.8% SD: 15.2% PD: 26.1%	Median OS: 9.5 mo (37 pts)	9 pts lost to FU. 37 pts underwent survival analysis.	^90^Y PET-CT done qualitatively after SIRT. Refractory cases had decreased survival (*p* = 0.00).
Filippi, L. et al. (2020) RS [32]	20 pts mixed	-	Mean: 1.6	Resin: 20	-	Mean OS: 12.5 mo	-	^90^Y PET-CT done qualitatively after SIRT.
Edeline, J. et al. (2020) PS [33]	41 pts (15 with multiple SIRT treatments) mixed	Median TD: 317 Median NTD: 87	-	Glass: 41	RECIST 1.1 at 3 mo RR: 39% * Control rate: 98%	Median OS: 22 mo Median PFS: 14 mo	Median FU: 36 mo 23 deaths.	SIRT and chemotherapy were concomitant.
Buettner, S. et al. (2020) RS [34]	114 pts refractory		Median Glass: 2.6 Resin: 1.6 (*p* = 0.00) Overall: 1.7	Resin: 92 Glass: 22	RECIST 1.1 at 6 mo (98 pts): Resin: PD: 26% SD: 69% PR: 3% Glass: PD: 30% SD: 45% PR: 25% (*p* = 0.00)	Median OS: 11 mo Resin: 11 mo Glass: 9 mo (*p* = 0.47) Median PFS: 5 mo Resin: 5 mo Glass: 3 mo (*p* = 0.85) Median liver-specific PFS: 6 mo	Median FU: Resin: 10 mo Glass: 14 mo 89 deaths.	One patient received both resin and glass microspheres and was excluded from analysis. Resin and glass microspheres had similar toxicity profiles (*p* = 0.35).
Bargellini, I. et al. (2020) RS [35]	81 pts mixed	Mean TD: 136.6	Mean: 1.4	Resin: 81	RECIST 1.1 at unspecified time (79 pts) CR: 5% PR: 36% SD: 41% PD:16% OR: 41% Control rate: 83%	Median OS: 14.6 mo	Median FU: 11.1 mo	OS and tumor response did not differ in naïve vs. chemo-refractory cases.
Azar, A. et al. (2020) RS [36]	22 pts mixed	-	Mean: 1.5	Resin: 22	-	Median OS: 9 mo	Median FU: 9.0 mo	-
White, J. et al. (2019) RS [37]	61 pts mixed		Median Resin: 1.5 Glass: 2.8	Resin: 45 Glass: 16		Median OS: 8.7 mo Median PFS: 2.8 mo Median LPFS: 3.1 mo	Median FU: 13.9 mo 33 deaths	No analysis was done based on type of microspheres used.
Galiè, F. et al. (2019) RS [38]	35 pts refractory	-	Median: 1.3	Resin: 35	RECIST 1.1 at 3 mo CR:0 PR: 25% SD: 47% PD: 28%	Median OS: 429 days	-	-
Bourien, H. et al. (2019) RS [39]	64 pts (20 with multiple SIRT treatments) mixed	Median TD: 269 Median NTD: 85	Median: 2.5	Glass: 64	RECIST 1.1 at unspecified time CR:0 PR: 15% SD: 61% PD: 24%	Median OS: 16.4 mo Median PFS: 7.6 mo	Median FU: 37.5 mo	OS was higher in those receiving radiation doses >260 Gy (*p* = 0.01).
Levillain, H. et al. (2019) PS [40]	58 pts refractory	Median BSA NTD: 26 TD: 38 Median partition NTD: 35 TD: 86	-	Resin: 58	-	Median OS: 10.3 mo	Median FU: 6.3 mo	-
Shaker, T. M. et al. (2018) RS [41]	17 pts (2 pts with multiple SIRT treatments) mixed	Mean TD, glass: 158.2 Mean TD, resin: 34.5 (*p* < 0.00)	-	Resin: 9 Glass: 8	-	Median OS: 33.6 mo LPFS: 4 mo glass: 2.4 mo resin: 15.6 mo (*p* = 0.46)	Median FU: 21.3 mo	^90^Y SPECT-CT done qualitatively after SIRT.
Reimer, P. et al. (2018) RS [42]	21 pts naïve	-	-	Resin: 21	RECIST at unspecified time CR:0 PR: 4.8% PD: 42.9% SD: 52.4%	Median OS: 15 mo	11 deaths	^90^Y PET-CT done qualitatively after SIRT.
Nezami, N. et al. (2018) RS [43]	10 pts naïve	Mean TD, glass: 205.7 Mean TD, resin:128.9 (*p* < 0.00) Mean NTD, glass: 42.4 Mean NTD, resin: 53.6 (*p* < 0.00)	Mean Glass: 2.7 Resin: 1.6 (*p* < 0.00)	Resin: 5 Glass: 5	-	-	-	Resin and glass microspheres had similar toxicity profiles (all *p* > 0.05).
Manceau, V. et al. (2018) RS [44]	35 pts 55 SIRT (20 pts with multiple SIRTs) naïve	Mean TD: 322 Mean NTD: 74	Mean: 2.6	Glass: 35	EASL at 3 mo CR: 47% PR: 49% SD: 4% PD: 0	Median OS: 28.6 mo Median PFS: 12.7 mo	Median FU: 20.7 mo	The mean TD for responding lesions (CR + PR) was 310 Gy.
Gangi, A. et al. (2018) RS [45]	85 pts 140 SIRT (40 pts with multiple SIRT treatments) mixed	Mean: 172.4 Median: 136.0	-	Glass: 85	RECIST at 3 mo (81 pts) CR: 0 PR: 6.2% SD: 64.2% PD: 29.6%	Median OS: 12.0 mo	Median FU: 9.8 mo	^90^Y PET-CT done qualitatively after SIRT. Median OS was significantly higher in pts with well-differentiated tumors (*p* = 0.01).
Swinburne, N. C. et al. (2017) RS [46]	29 pts (1 pt with multiple SIRT treatments) mixed	-	Mean: 1.6	Resin: 17 Glass: 12	RECIST 1.1 at 3 mo (26 pts) CR: 0 PR: 11.5% SD: 61.5% PD: 26.9% OR: 11.5%	Median OS: 9.1 mo Median TTP: 5.6 mo	Mean FU: 8.4 mo	^90^Y SPECT-CT done qualitatively after SIRT. OS correlated with the imaging-based response (*p* = 0.02). No analysis was done based on the type of microspheres used.
Jia, Z. et al. (2017) RS [47]	24 pts (3 with multiple SIRT treatments) refractory		Mean: 1.6	Resin: 24	mRECIST at 3 mo (22 pts): CR: 0 PR: 36.4% SD: 45.5% PD: 18.2% Control rate: 81.8% ^‡^	Median OS: 9.0 mo	Mean FU: 11.3 mo 19 deaths	
Akinwande, O. et al. (2017) RS [48]	25 pts 37 SIRTs (number of pts with multiple SIRT treatments unspecified) mixed	-	Median: 1.5	Resin: 11 Glass: 26	mRECIST at 1 mo (19 pts): CR: 5.2% PR: 0 SD: 57.8% PD: 36.8%	-	-	-
Soydal, C. et al. (2016) RS [49]	16 pts (2 with multiple SIRT treatments) mixed	-	Mean: 1.7	Resin: 16	RECIST at 3 mo OR: 31.2%	Median OS: 293 days	FU: 243 days 12 deaths	^90^Y SPECT-CT done qualitatively after SIRT.
Pieper, C. C. et al. (2016) RS [50]	26 pts mixed	-	Mean: 1.2	Resin: 26	-	-	-	Mean intended activity was 1.4 GBq; due to stasis, 86.9% was delivered.
Mosconi, C. et al. (2016) RS [51]	23 pts mixed	-	Mean: 1.5	Resin: 23	RECIST 1.1 at 3 mo (20 pts): CR:0 PR: 15.0% SD: 30.0% PD: 55.0% mRECIST at 3 mo (20 pts): CR: 5.0% PR: 40.0% SD: 15.0% PD: 40.0% EASL at 3 mo (20 pts): CR: 5.0% PR: 55.0% SD: 25.0% PD: 15.0%	Median OS: 17.9 mo	Median FU: 16.0 mo 17 deaths	OS was higher in treatment-naïve vs. refractory cases (*p* = 0.00).
Lam, M. G. E. H. et al. (2015) RS [52]	18 pts mixed	Median TD: 35 Median NTD: 24.9	-	Both (numbers not mentioned)	RECIST 1.1 at 3 mo OR: 18%	Median OS: 5.7 mo	-	-
Filippi, L. et al. (2015) PS [53]	17 pts mixed	-	Mean: 1.3	Resin: 17	PERCIST at 6 w CR: 0 PR: 82.3% SD: 17.6% PD: 0	Mean OS: 64.5 w Mean TTP: 28.9 w	-	-
Edeline, J. et al. (2015) RS [54]	24 pts naïve	Median TD: 256 Median NTD: 98	Median: 2.2	Glass: 24	RECIST at unspecified time: CR: 0 PR: 25.0% SD: 62.5% PD: 12.5% Control rate: 87.5%	Median OS was not reached. Median PFS: 10.3 mo	Median FU: 19.0 mo	Median PFS was higher with concomitant than with SIRT given before chemotherapy (*p* = 0.00)
Camacho, J. C. et al. (2014) PS [55]	21 pts refractory	-	-	Resin: 21	RECIST 1.1 at 1 mo: CR:0 PR: 4.7% SD: 76.2% PD: 19.1% mRECIST at 1 mo: CR:0 PR: 62.0% SD:19.0% PD:19.0% EASL at 1 mo: CR: 0 PR: 9.5% SD: 71.4% PD: 19.1%	Median OS: 16.3 mo	-	OS correlated with the modified target mRECIST and EASL scores at 3 mo (*p* = 0.00 for both).
Rafi, S. et al. (2013) PS [56]	19 pts 24 SIRT (4 with multiple SIRT treatments) refractory	-	Mean: 1.2	Resin: 24	RECIST at 3 mo: CR:0 PR: 10.5% SD: 68.4% PD: 21.0%	Median OS: 11.5 mo Median TTP: 4.8 mo	Median FU: 15 mo 12 deaths	-
Mouli, S. et al. (2013) PS [57]	46 pts 92 SIRT (32 pts with multiple SIRT treatments) mixed	Median: 90.9 Right Lobe of liver: 95.4 Left lobe of liver: 114.7	-	Glass: 46	WHO at unspecified time: CR: 0 PR: 23.9% SD: 71.7% PD: 2.1% EASL at unspecified time: CR: 9% PR: 64% PD: 0	No median OS	Median FU: 29 mo 39 deaths	-
Hoffman, R. T. et al. (2012) RS [58]	33 pts (1 with multiple SIRT treatments) mixed	-	Mean: 1.5	Resin: 33	RECIST at 3 mo: CR: 0 PR: 36.4% SD: 51.5% PD: 15.2%	Median OS: 22 mo Median TTP: 9.8 mo	-	-
Haug, A. R. et al. (2011) RS [59]	26 pts mixed	-	Mean: 1.7	Resin: 26	RECIST at 3 mo (23 pts): CR: 0 PR: 21.7% SD: 65.2% PD: 13.0%	Median OS: 11.7 mo	-	-
Saxena, A. et al. (2010) PS [60]	25 pts mixed	-	Mean: 1.7	Resin: 25	RECIST at 8.1 mo (23 pts): CR: 0 PR: 26.0% SD: 47.8% PD: 21.7%	Median OS: 9.3 mo	Median FU: 8.1 mo 2 deaths	-
Ibrahim, S. M. et al. (2008) PS [61]	24 pts mixed	Median: 105.1	-	Glass: 24	WHO at 1 mo (22 pts): CR: 0 PR: 27.2% SD: 68.1% PD: 4.5%	Median OS: 14.9 mo	Median FU: 17.7 mo 13 deaths	-

BSA, body surface area; CR, complete response; EASL, European Association for the Study of Liver Disease; FU, follow up; LPFS, liver-specific progression-free survival (the interval between treatment and disease progression or death from any cause); mRECIST, modified Response Evaluation Criteria in Solid Tumors; NTD, nontumor (liver) dose; OR, objective response (CR + PR); OS, overall survival; PD, progressive disease; PFS, progression-free survival; PR, partial response; PS, prospective study: pts, patients; RFS, recurrence-free survival; RR, response rate; RS, retrospective study; RECIST, Response Evaluation Criteria in Solid Tumors; SD, stable disease; SIRT, selective internal radiation therapy; TD, tumor dose; TTP, time to progression; ^90^Y PET-CT, ^90^Y positron emission tomography−computed tomography; ^90^Y SPECT-CT, ^90^Y single-photon emission tomography−computed tomography; WHO, World Health Organization criteria. * Six patients had extrahepatic cholangiocarcinoma; the rest had intrahepatic disease. There was no significant difference in OS based on the type of cholangiocarcinoma. ^†^ Mixed: treatment naïve and refractory cases ^‡^ Control rate: SD + CR + PR.

**Table 2:** Conference abstracts included in the study.

Author, Type of Study	Patient Population	Radiation Dosage, Gy	Activity, GBq	Microsphere Type: Number of Patients Treated	RECIST, WHO	Survival Outcomes	Follow-Up Information	Notes and Other Findings
Helmberger, T. et al. (2021) PS [62]	120 pts NM	-	Median (entire liver): 1.3 Right lobe of liver: 1.2 Left lobe of liver: 0.8	Resin: 120	-	Median OS: 14.7 mo Median PFS: 5.7 mo	24 mo	-
Lorenzoni, A. et al. (2020) RS [63]	23 pts 30 SIRTs (7 pts with multiple SIRT treatments) Mixed *	Mean TD: 309 NTD: 42.4	2.5	Glass: 30	mRECIST at unspecified time: CR: 3% PR: 3% SD: 87% PD: 7%	Median OS: 21 mo Median PFS: 9 mo	-	Mean TD stable disease lesions: 280 Gy, responding lesions (CR + PR): 384 Gy
Core, J. et al. (2020) RS [64]	32 pts 42 SIRT (Number of pts with multiple SIRT treatments unspecified) NM	Median TD: 253	-	-	mRECIST at 3 mo (36 pts): CR: 33.3% PR: 58.3% SD: 8.3% PD: 0	Median OS not reached.	Median FU: 10.9 mo	-
Pettinato, C. et al. (2019) RS [65]	35 pts mixed	Mean TD: 455.7 Mean NTD: 13.9	Mean: 1.4	Resin: 35	RECIST 1.1 at unspecified time: OR: 20% mRECIST at unspecified time: OR: 70% EASL at unspecified time: OR: 60%	Mean OS: 15.3 mo	4 deaths	-
Schatka, I. et al. (2017) RS [66]	33 pts refractory	-	Median: 1.8	Resin: 33	-	Median OS: 8 mo	-	-
Boni, G. et al. (2017) PS [67]	20 pts, 29 SIRT (3 pts with multiple SIRT treatments) mixed	-	Mean: 0.97	Resin: 29	-	Median TTP: 7.3 mo	-	-
Peterson, J. et al. (2010) RS [68]	9 pts NM	-	Median: 41 mCi ^†^	Resin: 9	(Criteria not mentioned) (7 pts) PR: 57%PD: 43%	OS at 9 mo: 89%	-	-

CR, complete response; NTD, nontumor (liver) dose; NM, (patient population in terms of previous treatments received) not mentioned; OS, overall survival; PD, progressive disease; PFS, progression-free survival; PR, partial response; PS, prospective study; RS, retrospective study; SD, stable disease; SIRT, selective internal radiation therapy; TD, tumor dose; TTP, time to progression. * Mixed: treatment naïve and refractory cases. ^†^ mCi: delivered activity measure.

**Table 3:** Studies in which SIRT and chemotherapy were given concomitantly to at least 1 patient.

Author, Year, Type of Study	No. of Patients Receiving Concomitant Chemotherapy/Total Number of Patients	Chemotherapy Regimen	Definition	Analysis
Depalo, T. et al. (2021) RS [25]	7/15	-	No definitions available. Concomitant chemotherapy was distinct from chemotherapy given before SIRT.	No significant difference in SIRT + chemo vs. SIRT alone, in terms of radiosensitivity (*p* value not available).
Paz-Fumagalli, R. et al. (2021) RS [26]	-	Cisplatin + gemcitabine	Concomitant chemotherapy was administered in 45 days before or after SIRT.	Unified results
Riby, D. et al. (2020) RS [29]	18/19	Cisplatin 50 mg/m^2^ + 5FU 400 mg/m^2^ Cisplatin 80 mg/m^2^ + capecitabine 1000 mg/m^2^ Gemcitabine 1000 mg/m^2^ + oxaliplatin 100 mg/m^2^ Cisplatin 25 mg/m^2^ + gemcitabine 1000 mg/m^2^ Oxaliplatin 85 mg/m^2^ + irinotecan 180 mg/m^2^ + 5FU 400 mg/m^2^ (Gemcitabine reduced to 300 mg/m^2^ for concomitant administration)	Concomitant chemotherapy was administered on the day before or the day after SIRT, but not on the same day.	SIRT + chemo vs. chemo vs. surgery: RFS and recurrence rate statistically the same (*p* = 0.28 and *p* = 0.21, respectively).
Edeline, J. et al. (2020) PS [33]	41/41	Cisplatin 25 mg/m^2^ + gemcitabine 1000 mg/m^2^ (Gemcitabine reduced to 300 mg/m^2^ for concomitant administration)	SIRT administered in cycle 1 for ICC (one hemi-liver) or in cycles 1 and 3 (both hemi-livers)	9 patients (22%) successfully down-staged to surgical resection.
Buettner, S. et al. (2020) RS [34]	4/114	5FU-based Cisplatin + gemcitabine	No definitions available. Concomitant chemotherapy was distinct from chemotherapy given before or after SIRT.	-
White, J. et al. (2019) RS [37]	7/61	-	No definitions available. Concomitant chemotherapy was distinct from chemotherapy given before or after SIRT.	-
Bourien, H. et al. (2019) RS [39]	33/64	Cisplatin 50 mg/m^2^ + 5FU 400 mg/m^2^ (bolus) Gemcitabine 1000 mg/m^2^ + oxaliplatin 100 mg/m^2^ Cisplatin 25 mg/m^2^ + gemcitabine 1000 mg/m^2^ Gemcitabine 1250 mg/m^2^ (Gemcitabine reduced to 300 mg/m^2^ for concomitant administration)	Chemotherapy was administered at most 3 months before SIRT. Chemotherapy administered more than 3 months before SIRT was considered induction chemotherapy.	Median PFS and median OS were not statistically different for the concomitant chemotherapy vs. induction vs. no chemotherapy groups (*p* = 0.90; *p* = 0.37, respectively).
Manceau, V. et al. (2018) RS [44]	35/35	Cisplatin 25 mg/m^2^ + gemcitabine 1000 mg/m^2^ Cisplatin 50 mg/m^2^ + 5FU 400 mg/m^2^ Gemcitabine 1000 mg/m^2^ + oxaliplatin 100 mg/m^2^ (Gemcitabine reduced to 300 mg/m^2^ for concomitant administration)	Chemotherapy administered at most 3 months before SIRT.	The exact tumor dose threshold for response in SIRT concomitant with chemotherapy could not be defined, but was below 158 Gy. 17 patients (49%) had successful downstaging.
Akinwande, O. et al. (2017) RS [48]	4/25	-	-	The disease control rate was not affected by concomitant chemotherapy administration (*p* = 0.99)
Pieper, C. C. et al. (2016) RS [50]	1/26	-	-	Unified analysis of SIRT application for different malignancies revealed concurrent chemotherapy is a predictor of stasis in SIRT (OR, 8.69; *p* = 0.00)
Edeline, J. et al. (2015) RS [54]	10/24	Cisplatin 50 mg/m^2^ + 5FU 400 mg/m^2^ Gemcitabine 1000 mg/m^2^ + oxaliplatin 100 mg/m^2^ Cisplatin 25 mg/m^2^ + gemcitabine 1000 mg/m^2^ (Gemcitabine reduced to 300 mg/m^2^ for concomitant administration)	Chemotherapy administered at most 3 months before SIRT.	The median PFS was higher in the concomitant chemotherapy group than in the induction group (*p* = 0.00).
Nezami, N. et al. (2019) PS [69]	5/5	Gemcitabine Dose level 1: 400 mg/m^2^ Dose level 2: 600 mg/m^2^ Dose level 3: 800 mg/m^2^ Dose level 4: 1000 mg/m^2^	Chemotherapy on one day before SIRT for 1-lobe treatment and 38 days before SIRT for 2-lobe treatment.	No gemcitabine-related toxicity on dose levels 1 and 2. All hepatic toxicities were on dose level 4. RECIST at 3 m: 100% stable disease.

NA: not available, OS: overall survival; PFS, progression-free survival; PS, prospective study; pts, patients; RECIST, Response Evaluation Criteria in Solid Tumors; RFS, recurrence-free survival; RS, retrospective study; SIRT, selective internal radiation therapy.

**Table 4:** Results of dosimetry verification studies.

Authors (Year)	No. of Patients, Microsphere Type	Dosimetry after Treatment	Delivered Dose (Gy)	Activity (GBq)	Dose-Response Analysis
Willowson KP, et al. (2021) [22]	18 pts Resin	^90^Y PET-CT	No mean tumor dose is reported for the whole study participants. Dose to normal liver is measured with ^99^mTc-mebrofenin scintigraphy and is only reported as unified results with some HCC patients.	Median: 1.5 Mean: 1.62	Average dose and minimum dose to 70% of lesion volume (D_avg_, D_70_) were not associated with lesion response (based on Total Lesion Glycolysis (TLG)) (*p* = 0.31, *p* = 0.60, respectively). TLG reduction of at least 50% was considered as significant response, with mean D_avg_ of 74 Gy for responding vs. 61 Gy for non-responding lesions (*p* = 0.29) and D_70_ 42 Gy vs. 27 Gy, respectively (*p* = 0.61).
Depalo, T. et al. (2021) [25]	15 pts Resin	^90^Y PET-CT	Mean TD: 93 NTD: 42 D_70_: 61	Mean: 1.16	Tumor Dose (Gy) showed positive effect on TTP on multivariate analysis (*p* = 0.05). D_70_ did not show any significant effect on TTP (*p* = 0.88)
Cheng B, et al. (2021) [27]	38 pts Glass & Resin	^90^Y SPECT-CT	Mean TD Resin: 78.9 Glass: 254.7	-	Tumor Dose (Gy) thresholds to reach at least 80% specificity for tumor objective response Mean TD: Resin: 78.9 Glass: 254.7 Minimum TD: Resin: 53.7 Glass: 149.1 Maximum TD: Resin: 162.9 Glass: 591 D_70_: Resin: 68.1 Glass: 221.7. Resin: Median OS of 20.2 m vs. 6.5 m for those with mean TD ≥75 Gy vs. less (*p* = 0.00). Glass: Median OS of 14.6 vs. 2.6 Gy for the mean TD ≥150 vs. less (*p* = 0.03)
Nezami, N. (2018) [43]	10 pts Glass & Resin	^90^Y SPECT-CT	Mean TD: Glass: 205.7, Resin:128.9 (*p* < 0.001) Mean NTD: Glass: 42.4, Resin:53.6 (*p* < 0.001) Tumor to normal parenchyma ratio: Glass: 4.9, Resin: 2.4 (*p* < 0.001)	Mean Glass: 2.75 Resin: 1.67 (*p* < 0.001)	-

NTD, nontumor dose; PS, prospective study; RS, retrospective study; SIRT, selective internal radiation therapy; TD, tumor dose; ^90^Y PET-CT, ^90^Y positron emission tomography−computed tomography; ^90^Y SPECT-CT, ^90^Y single-photon emission tomography−computed tomography.
